# Isolation and Identification of Soil Bacteria from Extreme Environments of Chile and Their Plant Beneficial Characteristics

**DOI:** 10.3390/microorganisms8081213

**Published:** 2020-08-10

**Authors:** Alexis Gaete, Dinka Mandakovic, Mauricio González

**Affiliations:** 1 Laboratorio de Bioinformática y Expresión Génica, Instituto de Nutrición y Tecnología de los Alimentos, Universidad de Chile, El Libano 5524, 7810000 Santiago, Chile; alex.ignacio@live.com; 2 Center for Genome Regulation, El Libano 5524, Santiago 7810000, Chile; 3 Programa de Doctorado en Ciencias Silvoagropecuarias y Veterinarias, Campus Sur Universidad de Chile. Santa Rosa 11315, 8820808 Santiago, Chile; 4 GEMA Center for Genomics, Ecology and Environment, Universidad Mayor, Camino La Pirámide 5750, 8320000 Santiago, Chile; dinka_slavja@hotmail.com; 5 Laboratorio de Genómica y Genética de Interacciones Biológicas (LG^2^IB). Instituto de Nutrición y Tecnología de los Alimento, Universidad de Chile. El Líbano 5524, 7810000 Santiago, Chile

**Keywords:** extreme environments, bacterial isolates, plant growth promoting bacteria

## Abstract

The isolation of soil bacteria from extreme environments represents a major challenge, but also an opportunity to characterize the metabolic potential of soil bacteria that could promote the growth of plants inhabiting these harsh conditions. The aim of this study was to isolate and identify bacteria from two Chilean desert environments and characterize the beneficial traits for plants through a biochemical approach. By means of different culture strategies, we obtained 39 bacterial soil isolates from the Coppermine Peninsula (Antarctica) and 32 from Lejía Lake shore soil (Atacama Desert). The results obtained from the taxonomic classification and phylogenetic analysis based on 16S rDNA sequences indicated that the isolates belonged to four phyla (Proteobacteria, Actinobacteria, Firmicutes, and Bacteroidetes), and that the most represented genus at both sites was *Pseudomonas*. Regarding biochemical characterization, all strains displayed in vitro PGP capabilities, but these were in different proportions that grouped them according to their site of origin. This study contributes with microbial isolates from natural extreme environments with biotechnological potentials in improving plant growth under cold stress.

## 1. Introduction

By definition, extreme environments meet conditions that represent a difficult life opportunity for any species [1]. The geography of Chile has many ecosystems that represent complex and extreme conditions in terms of salinity, humidity, UV radiation, temperature, pH, and heavy metals [2]. These conditions exert a selection pressure on the biodiversity of species, including microorganisms, which have evolved rapidly in these environments [3]. Two of the most extreme environments on Earth found in Chile include the Atacama Desert and Antarctica, which stand out for their extreme and fluctuating environmental conditions [4,5,6,7]. Antarctica is considered one of the most extreme sites in the world [8]. Here, the colonization of species and life is at its limit, not only because of the extreme low temperature and limited and sporadic availability of water, but also due to the constant cycles of freezing and thawing, continuous strong winds, and high UV radiation [9]. However, effects produced by deglaciation and climate change have allowed the colonization of plant species in soils that were previously covered by ice or snow [10,11,12]. Remarkably, plant species have managed to overcome these extreme environments and have been able to colonize and establish in these harsh scenarios. In fact, in the Coppermine Peninsula (Robert Island, Antarctic Peninsula), the Antarctic grass (*Deschampsia antarctica*) and the Antarctic carnation (*Colobanthus quitensis*) have been able to germinate and develop despite the extreme low temperature [13]. We believe that this has also produced an effect on the microbiological composition of the soil. On the other hand, the Atacama Desert is considered a cold and arid desert [14], but it presents important temperature fluctuations between day and night [5], which are even more significant in the highlands, where Lejía Lake is located. Here, it is possible to find native plant species such as *Calamagrostis crispa*, *Nassella nardoides,* and *Jarava frigida* [15,16] at an altitude of between 4000 and 4500 meters above sea level, which is attributed to the higher levels of precipitation and humidity in the area during the summer [15]. Studying how metabolic capacities of microorganisms are influenced by cold deserts, would help us understand how these microorganisms support different processes at the ecosystem level and develop new or better biotechnological tools based on bacteria [17].

Bacteria with the capacity to contribute to plant tolerance to different environmental vicissitudes have been described in extreme environments [18]. These bacteria are defined as Plant Growth Promoting (PGP) and are related to the physiological modifications of the plant that allow them to better manage the harsh environments through the exogenous production of auxin, stimulating the elongation of adventitious roots or through the 1-aminocyclopropane-1-carboxylic acid (ACC) deaminase activity, which intervenes in the production of ethylene, inhibiting the response to senescence. Other mechanisms in which PGP bacteria also participate is in nitrogen fixation, phosphate solubilization, or siderophore production, all of which relate to the increase in nutrient availability in the soil [19]. Research has been carried out based on the use of PGP in agriculture to improve the yield of fruit trees, vegetables, and cereals [20] such as potato [21], wheat [22], soybean [23], apple [24], and tomato [25], among others [26,27]. However, the isolation and application of PGP from extreme conditions represents a rather unexplored scenario [28]. In this study, we isolated, identified, and characterized 71 soil bacteria from desert (Coppermine Peninsula and Lejía Lake shore), highlighting their similarities and differences attributed to their taxonomy, plant beneficial traits, and origin.

## 2. Materials and Methods

### 2.1. Sample Collection and Processing

Two extreme Chilean environments were selected for this study. The first site corresponds to Lejía Lake (23°30′ S 67°41′24″ W) in the Atacama Desert, and the second site corresponds to the Coppermine Peninsula on Robert Island, Antarctic Peninsula (62°22′43″ S 59°42′21.9″ W). From both sites, approximately 100 g of bulk soil samples were taken in quadruplicate at 10 cm depth to avoid contamination with other surfaces. Each sample was placed in sterile plastic bags and immediately stored in cold (IcePack). Samples were used for the preparation of Soil Extract Medium (SEM) as a matrix for bacterial isolation and for physical–chemical analysis. 

Soil samples were taken under similar environmental conditions (during the summer after the rainy season) but in different years. Sampling in the Atacama Desert was conducted in April 2014 and that in the Antarctic Peninsula was performed in January 2019 by the ECA-55 (LV Antarctic Scientific Expedition) of INACH.

### 2.2. Physical–Chemical Soil Analysis

Temperature was recorded using a digital thermometer (Long-Stem, Thomas Traceable, Federal Government, USA) and geolocation was obtained using a Global Positioning System (GPS) equipment (64sx, Garmin, Lenexa, KS, USA). Soil pH was achieved by mixing soil:distilled water (1:1 *p*/*v*) and measurement was obtained using a pH-Meter (Orion 3, Thermo Scientific, Waltham, MA, USA). The elemental composition of the soil was measured by Total Reflection X-ray Fluorescence Spectrometry (TXRF), using Bruker S2 PICOFOX equipment [29] following previously published protocol [5,16]

### 2.3. Culture Conditions and Bacterial Isolation

After field sampling, the isolation of bacteria from the collected samples was performed. Two culture media were used in both sites to obtain bacterial isolates: SEM and Luria–Bertani (LB) [30]. Briefly, SEM was prepared by mixing agar with an aqueous soil fraction (soil:distilled water, 1:1 *p*/*v*). This fraction was obtained using 80 g of soil mixed with 80 mL of distilled water. This mix was agitated for 1 hour and then decanted overnight. The soluble fraction was used to hydrate the agar (1.5%), and finally, the mixture was autoclaved and plated. LB was prepared using 10 g of yeast extract, 5 g of peptone, 10 g of sodium chloride, 10 g of agar, and 1 L of distilled water. To obtain bacterial isolations, the protocol described by Mandakovic et al. [6] previously used to isolate bacteria from soil samples in the Lejía Lake was followed. Briefly, 2 g of soil was mixed into 2 mL of sterile Phosphate Buffered Saline (PBS) for 2 h. After that time, the tubes were centrifuged for 5 min at 5000 rpm, and 100 µL of supernatant was seeded into each of the culture media (SEM and LB) in triplicate and incubated at 30 °C. Additionally, in order to increase the possibilities of isolating specific bacteria from cold areas, samples from the Coppermine Peninsula were also incubated at 16 °C for seven days in plates with SEM and LB media. Morphologically different colonies were isolated and maintained in LB medium. All strains classified as unique in the culture were stored in glycerol at −80 °C, creating a culture collection of bacteria from these extreme environments.

### 2.4. Taxonomical Assignation

The DNA of each strain was extracted using a DNeasy Blood & Tissue Kit (QIAGEN, Hilden, Germany). The 16S rDNA region was amplified using the 27F and 1492R primers. The PCR mix contained 12.5 µL of Mastermix Go Taq Promega, 8.5 µL of nuclease-free water, 1 µL of each primer, and 2 µL of DNA. Thermal cycling followed the following steps: 10 min at 95 °C, 30 cycles of 95 °C for 60 s, 58 °C for 30 s, and 72 °C for 60 s, and a final extension at 72 °C for 10 min. PCR products were kept at 4 °C until use. PCR products were visualized in 1% (*w*/*v*) agarose gel electrophoresis and sequenced (Macrogen, Inc, Geumcheon-gu, Seoul, Korea). Taxonomy was defined using the EzBioCloud (16S database), and sequences were deposited in the NCBI Genbank database. Accession numbers can be found in Table S1. 

### 2.5. Phylogenetic Analysis

For the construction of the Neighbor Joining Tree, all 16S rDNA sequences obtained were trimmed according to the quality of the sequencing using CLC software (QIAGEN Bioinformatics, Hilden, Germany). Multiple cluster alignment and phylogenetic analysis were performed on MEGA software (v. 7.2) based on the neighbor binding method using a 1000 repetition bootstrap to evaluate statistical support [31].

### 2.6. In Vitro Identification of Plant Growth Promoting Traits

To biochemically classify bacteria with beneficial plant traits, five capacities were evaluated for each strain: siderophore production, auxin (AIA) production, nitrogen fixation, 1-aminocyclopropane-1-carboxylic acid (ACC) deaminase activity, and phosphate solubilization, as described in Maza et al. [30]. The production of siderophores was determined using the method described by Schwyn and Neilands [32]. Briefly, using chrome azurol agar (CAS), colonies that exhibited a yellow halo after proliferation were classified as positive for siderophore production. AIA production was evaluated by a colorimetric method. Isolates were cultured in Tryptic Soy Broth (TSB) media supplemented with 0.1% L-tryptophan as a precursor to AIA. Using Salkowski’s reagent [33], the TSB media turned from yellow to violet coloration according to the concentration of AIA present in the medium, which was compared to a standard curve of indol-3-acetic acid (Merck, Burlington, MA, USA) ranging from 5 μg/mL to 180 μg/mL. Nitrogen fixation was determined using nitrogen-free medium (NFM). Isolates were incubated for seven days and bacterial growth evidenced atmospheric nitrogen fixation [34]. ACC deaminase activity was measured by culturing bacteria with 3 mM of ACC (Merck, Burlington, MA, USA) as the only source of nitrogen [35]; thus, bacterial growth evidenced the use of this metabolite. Finally, the phosphate solubilization capacity was determined by the presence of a halo surrounding the colonies on Pikovskayas agar medium after four days of culture [36].

### 2.7. Similarity/Dissimilarity Test

The similarity evaluation was performed using Past software (v. 4.03) through a multivariate analysis with a matrix that included each isolate, genus, and origin site. The algorithm used was the unweighted pair group method with arithmetic mean (UPGMA) and Jaccard similarity index.

## 3. Results and Discussion

### 3.1. Environmental and Soil Characterization of the Study Sites

**Figure 1:**
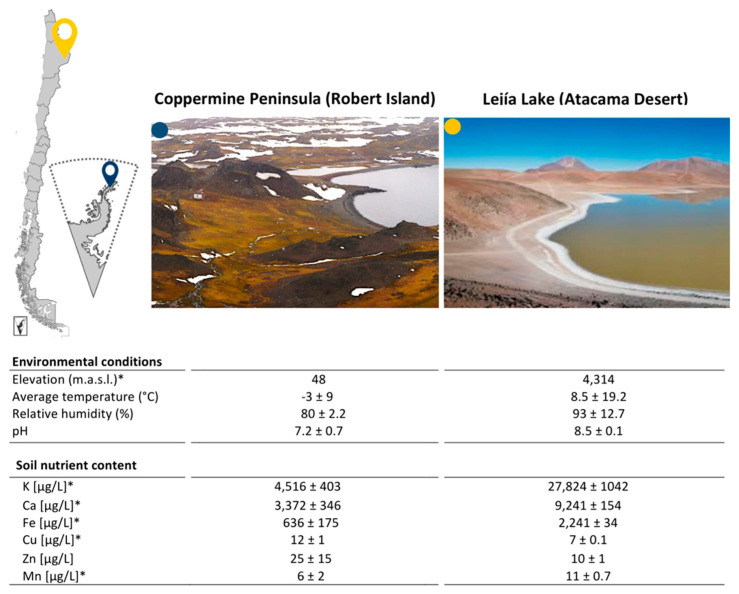
Extreme environments as sampling sites. Blue (Coppermine Peninsula) and yellow (Lejía Lake) icons indicate their specific locations on the map. The physicochemical parameters of both sites are described in the table. * *t*-test < 0.05.

Two Chilean desert sites were selected for this study: Lejía Lake, located in the north of Chile, specifically in the highlands of the Atacama Desert, and Coppermine Peninsula, located in the extreme-south of the country, which is part of Robert Island in the Antarctic Peninsula. To characterize the physical–chemical features of these two Chilean deserts, we performed environmental measurements during sampling and subsequently identified soluble elements in the soil through TXRF. We observed that despite being obtained from distant environments, the environmental conditions of both soil sampling sites were not significantly different in temperature, pH, and humidity at the time of sampling (Figure 1). We expected that there would be no differences between these parameters because the sampling was performed under similar environmental conditions in both sites, after periods of constant rain, snow, and extensive cold, followed by thawing and the colonization of plant species [10,16]. However, when evaluating the chemical elements present in the soil, K, Ca, Fe, and Mn were significantly higher in Lejía Lake shore soil than in the Coppermine Peninsula. These values may have a biological explanation attributed to the activity and constant fumaroles of the Lascar volcano that is located immediately above Lejía Lake. These elements, among others, have been identified and quantified through Energy-Dispersive Spectroscopy (EDS) from samples of fumaroles collected in 2012 from the Lascar Volcano [37]. On the other hand, Cu measured in soil from Lejía Lake was double the amount measured from the Coppermine Peninsula, which is an effect that could be attributed to the structure of the rock that makes up the peninsula and to mechanical weathering according to the biological processes at this site [38]. In fact, this peninsula has its name due to the evident presence of Cu in the rocks [39]. 

### 3.2. Taxonomic Classification and Biochemical Characterization of Isolates

**Figure 2:**
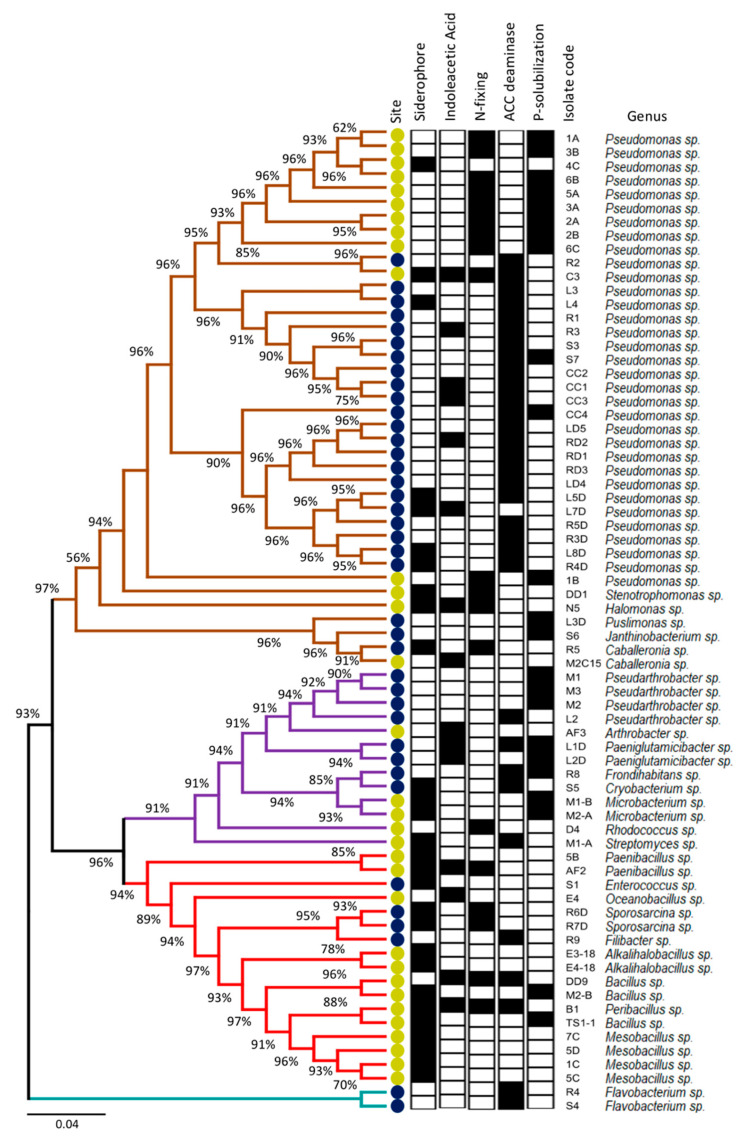
Phylogenetic analysis based on 16S rDNA gene sequences and characterization of plant beneficial traits of soil bacterial isolates. The colored branches represent different phyla: Proteobacteria (brown), Actinobacteria (purple), Firmicutes (red), and Bacteroidetes (blue). The colored circles represent different sampling sites: Lejía Lake (yellow) and Coppermine Peninsula (blue). Positive (black box) and negative plant beneficial traits (white box). Bar, 0.04 substitutions per position.

Two culture media (SEM and LB) were used to isolate strains from Coppermine Peninsula and Lejía Lake soils (for details, see Materials and Methods). A total of 39 bacteria were isolated from Coppermine Peninsula and 32 were isolated from Lejía Lake (Table S1). To taxonomically identify all isolated bacteria, the complete 16S rDNA gene was amplified and sequenced. The phylogenetic tree generated using these sequences (Figure 2) showed that the most abundant phyla in both sites corresponded to Proteobacteria and Actinobacteria, which were represented in similar quantities regardless of origin. On the other hand, Firmicutes was more represented in Lejía Lake, whereas strains related to Bacteroidetes were only isolated from the Coppermine Peninsula. 

When compared to other studies carried out that isolated bacteria from soils under extreme environmental conditions in different parts of the world, our results were consistent with the isolation of mainly Proteobacteria, Firmicutes, Bacteroidetes, and Actinobacteria [40,41,42]. The most representative phylum corresponded to Proteobacteria at both sampling sites. A study conducted by Maza et al. [30] that analyzed the cultivable fraction of bacteria from Atacama Desert soil reported this same result, and a similar study developed by Gao et al. [43] through the isolation of bacteria from soil samples in arid and semi-arid sites in China described that Proteobacteria, Firmicutes, and Actinobacteria (in that order) were the most abundant phyla, while Bacteroidetes was less represented. The authors observed that this phylum seems to be more frequent in soils with better nutritional conditions; however, the phylum has also been commonly found in soils containing high concentrations of copper [44], which is consistent with the results presented above. In addition, studies carried out on other islands of the Antarctic Peninsula, specifically on Windmill Island [45] and King George Island [46], also reported that the most abundant phylum obtained from isolated bacteria corresponded to Proteobacteria.

Regarding genera isolated, *Pseudomonas* was the most represented genus in our study, with 22 isolates from Coppermine Peninsula (56.4%) and 11 isolates from Lejía Lake (34.4%). We also observed the presence of less represented but specific genera in Lejía Lake, such as *Stenotrophomonas* (DD1), *Halomonas* (N5), *Arthrobacter* (AF3), *Microbacterium* (M1-B and M2-A), *Rhodococcus* (D4), *Streptomyces* (M1-A), *Paenibacillus* (5B and AF2), *Oceanobacillus* (E4), *Alkalihalobacillus* (E3-18 and E4-18), *Bacillus* (DD9, M2-B and TS1-1), *Peribacillus* (B1), and *Mesobacillus* (7C, 5D, 1C and 5C). Regarding the Coppermine Peninsula, exclusive genera found in the isolates from this site were *Pusillimonas* (L3D), *Janthinobacterium* (S6), *Pseudarthrobacter* (M1, M2, M3, and L2), *Paeniglutamicibacter* (L1D and L2D), *Frondihabitans* (R8), *Cryobacterium (S5)*, *Enterococcus* (S1), *Sporosarcina* (R6D and R7D), *Filibacter* (R9), and *Flavobacterium* (R4 and S4). Romaniuk et al. [46] similarly reported that *Pseudomonas* was one of the most abundant genera isolated from the soil from King George Island; however, another abundant genus was *Psychrobacter*, from which we obtained no isolates. This may be due to the difference in culture media used in each study. As for the rest of the studies related to microorganisms isolated from Antarctic soils, they focused more on the search for fungi [8,47,48,49] or endophytic bacteria [50], and not on bulk soil bacteria.

Two bacteria of the genus *Pseudomonas* (isolate C3) and *Peribacillus* (isolate B1) were isolated from the Lejía Lake, which had four positive PGP capacities, while in the Coppermine Peninsula, the soil isolate with the highest number of PGP capacities corresponded to the genus *Paeniglutamicibacter* (isolate L1D) with three PGP capacities out of the five evaluated. In general, Antarctic strains had less PGP traits than Lejía Lake isolates. Most of the Coppermine Peninsula strains possessed ACC deaminase activity, while the strains from Lejía Lake were more heterogeneous in terms of their PGP capabilities. The fact that a high percentage of bacteria present in the soil had at least one PGP capacity despite being isolated from bulk soil is consistent with a study carried out in Namibia Desert, in which a higher percentage of bacteria with PGP activities was isolated from bulk soil rather than from rhizospheres, proposing these strains as potential biofertilizers for arid areas [51]. The genus *Sporosarcine* was only represented in the Coppermine Peninsula, presenting two of the five PGP capacities evaluated. In this context, Yadav et al. [52] described a series of bacteria isolated from the Himalayan desert, declaring *Sporosarcine* to be a potent PGP candidate to use in low-temperature crops. Additionally, *Halomonas* genus was part of the set of bacteria isolated and characterized as plant beneficial traits from Lejía Lake. This genus was also isolated and characterized in the desert of southern Tunisia, and the genus was proposed as a potential plant growth promoter under salinity and drought conditions [53]. Other authors [54,55] have suggested that PGP bacteria isolated from extreme environments can be used as inoculants under similar conditions from where they were isolated; thus, bacteria isolated from a cold desert may be used to counteract cold stress, while bacteria isolated from arid conditions can be used to reduce drought effects [56]. 

### 3.3. Similarity/Dissimilarity of Soil Isolates

**Figure 3:**
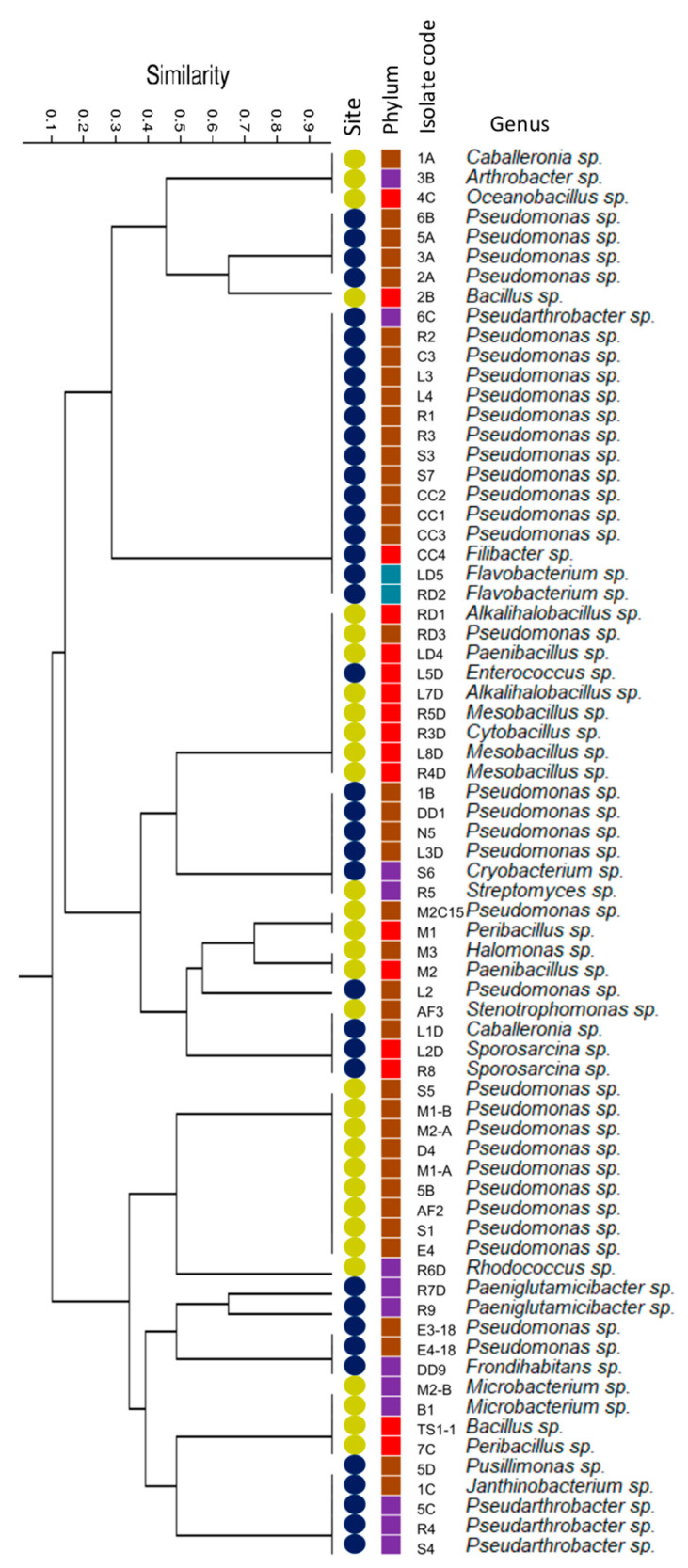
Similarity analysis through the Jaccard index. The colors of the circles represent different sampling sites: (yellow) Lejía Lake and (blue) Coppermine Peninsula. The colored box represents different Phyla. Proteobacteria (brown), Actinobacteria (purple), Firmicutes (red), and Bacteroidetes (light blue).

To evaluate the similarity among all the isolates, we calculated the Jaccard distance considering the origin of the sample, their taxonomic identification, and their PGP capacities. Figure 3 shows that each strain is specifically grouped according to the place of origin and its PGP capabilities. Phyla and genera are rearranged and randomly distributed in the dendrogram. The group with the most isolates corresponded to the strains that possessed ACC deaminase capacity only, all belonging to Coppermine Peninsula soil. The second most represented group contained only bacteria isolated from Lejía Lake soil, which had the PGP characteristics of nitrogen fixation and phosphate solubilization.

We expected that the bacteria isolated in the two sampling sites would have equivalent PGP capabilities, considering that both deserts have similar environmental conditions and also share taxonomic groups. Therefore, we estimated that when performing a multivariate test, the main factors that would cluster together would be taxonomy and plant beneficial traits, regardless of the origin of the sample. Nevertheless, at the time of conducting the similarity test using the Jaccard index, taxonomy was irrelevant, and isolates were grouped by their PGP capabilities, which also coincided with their isolation site. This could be explained by the fact that despite having similar environmental conditions during sampling, sites at other times of the year are completely different, which could impact site-specific microbiological adaptations and PGP characteristics. For example, for most of the year, the Coppermine Peninsula is covered with snow [10], while in Lejía Lake, it only snows for a few weeks a year and only in winter [30].

Although further investigations should be done, including the addition of more isolates, we think that these differences could be due to nutritional factors (including K, Ca, Fe, Cu, and Mn) (Figure 1), suggesting a tight connection between the PGP capacities of bacterial isolates and the environment [5]. However, there are differences among the plant beneficial traits in bacterial isolates located in different regions of the world, making it difficult to separate historic and phylogeographic effects from local features of soil. Antarctica is considered one of the most extreme sites in the world, where different conditions and environments not found elsewhere can be studied [8]. Here, the colonization of species and life is at its limit, not only because of the cold and low availability of water, but also due to the constant cycles of freezing and thawing, continuous strong winds, and high UV radiation [9]. Additionally, effects produced by deglaciation and climate change have allowed the colonization of plant species in soils that were previously covered by ice or snow [6,7]. We believe that this has also produced an effect on the microbiological composition of the soil. On the other hand, the Atacama Desert is considered a cold and arid desert [14], but it presents important temperature fluctuations between day and night [5], which are even more significant in the highlands, where Lejía Lake is located. It is also possible to observe vegetation of different herbaceous species at an altitude of between 4000 and 4500 meters above sea level, which is attributed to the higher levels of precipitation and humidity in the area. Lower altitudes correspond to the beginning of the absolute desert [15]. Studying desert soils from these two sites in relation to the metabolic capacities of microorganisms could help us understand how they sustain life in different extreme conditions, which may also impact new or better biotechnological tools [17].

Finally, Jorquera et al. [1] declare that species present in extreme environments are modifying their distribution and abundance due to climate change, and as a result, the microbial communities that inhabit these places represent a biotechnological tool with the capacity to solve current problems in agriculture, focused on improving the tolerance of crops in drought, floods, heat stress, and saline levels, among others. Therefore, bacteria with plant growth capacities obtained from these extreme desert environments are an unexplored source of novel biological resources, with enormous potential for biotechnology and its industry.

Extreme environments have been studied in various settings due to the interesting capacities developed by the different species that inhabit them [1]. In particular, it has been shown that bacteria are the first organisms to colonize these environments through metabolic adaptations [57], which increase the possibility of colonization by species that act as biological stabilizers (fungi, lichens, and mosses), which could also be an important niche for seed germination or the establishment of plant species [58]. Generally, bacteria with PGP capabilities have been isolated from crops with agronomic interest under stable environmental conditions [59] in order to improve crop yield [60,61], while PGP bacteria isolated from extreme environments are relatively unexplored [62]. In an era of climate change, the identification, isolation, and characterization of bacteria with PGP properties that come from extreme environments that may favor plant growth under abiotic stress conditions represent excellent and novel biotechnological tools to fight future harsh conditions [30,63]. Furthermore, soil for vegetable cultivation is increasingly limited, which is a problem for world food security; therefore, the use of these bacteria involves increasing the availability of nutrients in the soil and improving the tolerance, productivity, and yield of plants of agronomic interest [64,65,66].

The ability of bacteria to adapt and survive in cold environments attracts many researchers interested in solving this adaptive attribute and also in developing new or better biotechnological tools. Bacteria of the genus *Arthrobacter*, *Bacillus*, *Janthinobacterium*, *Pseudomonas*, and *Sporosarcine*, among others, have been described as having different mechanisms associated with cold stress tolerance, such as cold acclimation proteins (Caps) and antifreezing proteins (AFPs) that protect the specific cytoplasmic and extracellular components involved in cold adaptation [55]. In addition, a study by Zubair et al. [67] showed that the *Bacillus* genus presents mechanisms of response to cold stress through the formation of biofilms, the production of Reactive Oxygen Species (ROS), and also through the expression of the *acdS* gene, which is related to the ACC deaminase activity committed to the PGP capacity. 

## Supplementary Materials

The following are available online at https://www.mdpi.com/2076-2607/8/8/1213/s1, Table S1. Taxonomic and molecular information of bacteria isolated from desert soils.
